# Associations between an inflammatory diet index and severe non-alcoholic fatty liver disease: a prospective study of 171,544 UK Biobank participants

**DOI:** 10.1186/s12916-023-02793-y

**Published:** 2023-04-03

**Authors:** Fanny Petermann-Rocha, Michael D. Wirth, Jirapitcha Boonpor, Solange Parra-Soto, Ziyi Zhou, John C. Mathers, Katherine Livingstone, Ewan Forrest, Jill P. Pell, Frederick K. Ho, James R. Hébert, Carlos Celis-Morales

**Affiliations:** 1 School of Cardiovascular and Medical Health, BHF Glasgow Cardiovascular Research Centre, University of Glasgow, Glasgow, G12 8TA UK; 2 Centro de Investigación Biomédica, Facultad de Medicina, Universidad Diego Portales, Santiago, Chile; 3 College of Nursing, University of South Carolina, Columbia, USA; 4 Department of Epidemiology and Biostatistics and Cancer Prevention and Control Program, Arnold School of Public Health, University of South Carolina, Columbia, USA; 5 Faculty of Public Health, Chalermphrakiat Sakon Nakhon Province Campus, Kasetsart University, Sakon Nakhon, Thailand; 6 Department of Nutrition and Public Health, Universidad del Bío-Bío, Chillan, Chile; 7 School of Health and Wellbeing, University of Glasgow, Glasgow, UK; 8 Human Nutrition Research Centre, Centre for Healthier Lives, Population Health Sciences Institute, Newcastle University, Newcastle Upon Tyne, NE2 4HH UK; 9 Institute for Physical Activity and Nutrition, School of Exercise and Nutrition Sciences, Deakin University, Geelong, VIC 3220 Australia; 10 Department of Gastroenterology, Glasgow Royal Infirmary, University of Glasgow, Glasgow, UK; 11 Human Performance Laboratory, Education, Physical Activity and Health Research Unit, Universidad Católica del Maule, Talca, Chile

**Keywords:** Diet, Non-alcoholic fatty liver disease, Incidence, Inflammation, Prospective studies

## Abstract

**Background:**

Although non-alcoholic fatty liver disease (NAFLD) is linked to inflammation, whether an inflammatory diet increases the risk of NAFLD is unclear. This study aimed to examine the association between the Energy-adjusted Diet Inflammatory Index (E-DII) score and severe NAFLD using UK Biobank.

**Methods:**

This prospective cohort study included 171,544 UK Biobank participants. The E-DII score was computed using 18 food parameters. Associations between the E-DII and incident severe NAFLD (defined as hospital admission or death) were first investigated by E-DII categories (very/moderately anti-inflammatory [E-DII <  − 1], neutral [E-DII − 1 to 1] and very/moderately pro-inflammatory [E-DII > 1]) using Cox proportional hazard models. Nonlinear associations were investigated using penalised cubic splines fitted into the Cox proportional hazard models. Analyses were adjusted for sociodemographic, lifestyle and health-related factors.

**Results:**

Over a median follow-up of 10.2 years, 1489 participants developed severe NAFLD. After adjusting for confounders, individuals in the very/moderately pro-inflammatory category had a higher risk (HR: 1.19 [95% CI: 1.03 to 1.38]) of incident severe NAFLD compared with those in the very/moderately anti-inflammatory category. There was some evidence of nonlinearity between the E-DII score and severe NAFLD.

**Conclusions:**

Pro-inflammatory diets were associated with a higher risk of severe NAFLD independent of confounders such as the components of the metabolic syndrome. Considering there is no recommended treatment for the disease, our findings suggest a potential means to lower the risk of NAFLD.

**Supplementary Information:**

The online version contains supplementary material available at 10.1186/s12916-023-02793-y.

## Background

Non-alcoholic fatty liver disease (NAFLD) — along with its progressive form non-alcoholic steatohepatitis (NASH) — remains the most common chronic liver disease worldwide, affecting around a quarter of the adult population [1, 2]. The condition is characterised by excessive hepatic fat accumulation and is directly associated with metabolic risk factors like insulin resistance [3]. In 2019, the economic burden of the disease was estimated at €35 billion/year in Europe [4]. Unfortunately, both its prevalence and economic burden are likely to rise because the condition is associated with other highly prevalent non-communicable diseases, notably obesity and type 2 diabetes [5].

Because there are currently no successful pharmacological treatments for NAFLD [3, 6], there is an urgent need to identify modifiable risk factors that might prevent or delay its development. Low physical activity levels, low physical capability markers (such as low grip strength and low muscle mass), overweight and unhealthy diets are among the behavioural factors that might be the basis for potential strategies to prevent or treat NAFLD through intervention programmes [3, 6–8]. Diet is a recognised modifiable risk factor for NAFLD [6, 9–12] and various components of the diet, including energy restriction, macronutrient composition and fructose intake, have been identified in the aetiology of NAFLD [13–19]. Despite this, the specific dietary pattern that predisposes to NAFLD risk is still unclear. Even though NAFLD has been linked to inflammation, there is insufficient evidence on whether pro-inflammatory diets are associated with a higher risk of NAFLD.

The description of the original Diet Inflammatory Index (DII®) was published in 2009 [20, 21] as a tool to categorise individuals’ diets. In the last decade, the index has been used in over 750 studies to explore the associations of anti- or pro-inflammatory diets with morbidity and mortality [22–25]. Although other diet-quality scores/indices exist and contribute to understanding the role of dietary patterns in people’s health [26–29], the DII has the advantage of having been validated against circulating concentrations of C-reactive protein and other markers of systemic inflammation [20, 21, 30].

Despite the importance of this issue, there is only one prospective study, conducted in a non-British population, that has examined the association between an inflammatory diet and NAFLD [31]. The ATTICA study was conducted on a small but representative sample of 3042 Greek adults without pre-existing cardiovascular conditions [31]. That study used a modified version of the DII, which was referred to as the Dietary Anti-Inflammation Index (D-AII). Results from that study showed an inverse relationship with NAFLD; however, four equations were used as a proxy for NAFLD rather than a doctor diagnosis. In addition, the D-AII has not been standardised to a global comparative standard; therefore, results are not easily compared across populations, as can be done using the DII. Considering these limitations, the present study aimed to examine the association between an Energy-adjusted DII (E-DII) score and severe NAFLD in middle-aged and older adults from the UK Biobank prospective cohort study.

## Methods

UK Biobank is a prospective cohort that enrolled over 500,000 participants aged 37–73 years from the general UK population at baseline (5.5% response rate) [32]. In brief, between 2006 and 2010, participants attended one of 22 assessment centres across Scotland, England and Wales [33, 34]. All participants completed a touch-screen questionnaire, had physical measurements taken and provided blood, urine and saliva samples at baseline. More information about the UK Biobank protocol can be found online (http://www.ukbiobank.ac.uk).

### Diet Inflammatory Index

Dietary intake was measured using the Oxford WebQ, a web-based 24-h dietary assessment tool that collects information on 206 foods and 32 beverages consumed during the past 24 h [35, 36]. Energy and nutrient intake were calculated using *McCance and Widdowson’s The Composition of Food*, 5th edition [37]. Information from the dietary assessment tool was collected according to the previous day’s intake using questions such as: “did you have any of these yesterday?” or “how much of the following did you drink yesterday?” For this study, the average of five 24-h recalls was used (the information was collected between April 2009 [first instance] and June 2012 [last instance] as described on the UK Biobank webpage: https://biobank.ndph.ox.ac.uk/showcase/field.cgi?id=26008). People with unfeasible energy intake were excluded based on Henry’s equation.

The DII proposed by Shivappa et al. in 2014 included 45 different dietary factors (i.e. food parameters consisting of nutrients and whole foods) and their positive (pro-inflammatory) or negative (anti-inflammatory) effects, as described elsewhere [21]. Following the scoring algorithm proposed by Shivappa et al. [21], 18 foods and nutrients available in the UK Biobank dataset were included to create the DII: alcohol, carbohydrate, fibre, folate, saturated fat, polyunsaturated fat, protein, total fat, vitamin B12, vitamin B6, iron, magnesium, vitamin C, vitamin E, tea, garlic, onions and total energy. These 18-food parameter-specific DII scores were summed to obtain the overall DII score. To compute the E-DII score, the following steps were carried out: (1) instead of absolute intake reported, intake was expressed per 1000 kcal/day, and (2) the global comparative database also used energy-adjusted values, i.e. per 1000 kcal/day. The E-DII was used for all the analyses. The advantage of the E-DII over the original DII is that it accounts for inter-individual differences in energy intake [30]. The E-DII was used in place of the DII as it produced better goodness of fit statistics.

The E-DII score was used as a continuous variable and classified into three categories — from anti-inflammatory (scores down to − 4.39) to pro-inflammatory (scores up to 3.45) — as follows: (i) very/moderately anti-inflammatory (< − 1), (ii) neutral (≥ − 1 to ≤ 1) and (iii) very/moderately pro-inflammatory (> 1). A similar classification of the E-DII was previously used [38].

### Severe NAFLD

Severe NAFLD was defined as hospitalisation or death due to NAFLD or NASH and was ascertained from the linked hospital and death databases. The date and cause of death were obtained from death certificates held by the National Health Service (NHS) Information Centre (England and Wales) and the NHS Central Register Scotland (Scotland). Dates and causes of hospital admissions were identified via record linkage to Health Episode Statistics (England and Wales) and the Scottish Morbidity Records (SMR01) (Scotland). Details of the linkage procedure can be found at http://content.digital.nhs.uk/services. Hospital admissions data were available until September 2021 in England, July 2021 in Scotland and February 2018 in Wales. Therefore, incident event models were censored on these dates or the date of death if this occurred earlier. Mortality data were available until the end of October 2021. Therefore, follow-up was censored on these dates. Only the first event was taken for all analyses.

Using the International Classification of Diseases, 10th revision (ICD-10), and the latest Expert Panel Consensus Statement [39], NAFLD was defined as ICD-10 K76.0 (fatty [change of] liver, not elsewhere classified) and K75.8 (NASH, other specified inflammatory liver diseases).

### Covariates

Age at baseline was determined from dates of birth and baseline assessment. Sex was self-reported at baseline. Deprivation (area-based socioeconomic status) was derived from the postcode of residence, using the Townsend score [40]. Ethnicity was self-reported and categorised as white and others. Self-reported smoking status was categorised as never, former or current smoker. The components of the metabolic syndrome (central obesity, hyperglycaemia/diabetes, high blood pressure/hypertension, low HDL and high triglyceride) were ascertained using baseline data. Central obesity was defined as a waist circumference higher than 88 cm in women and 102 cm in men. Hyperglycaemia/diabetes was defined as fasting glucose ≥ 5.6 mmol/l or a self-report of a physician diagnosis of diabetes. High blood pressure/hypertension was defined as a systolic blood pressure ≥ 130 mm Hg and/or a diastolic blood pressure ≥ 85 mm Hg or a self-report of a physician diagnosis of hypertension. High triglyceride was defined as ≥ 1.7 mmol/l and low HDL-cholesterol as < 1.3 mmol/l in women and < 1.0 mmol/l in men [41–43]. Arthritis, inflammatory bowel disease and asthma were self-reported at the baseline appointment and codified as having or not having an inflammatory disease. Finally, the level of physical activity was self-reported using the International Physical Activity Questionnaire short form [44]. Additional information on the measurements is available on the UK Biobank website (http://www.ukbiobank.ac.uk).

### Statistical analyses

Descriptive baseline characteristics by the E-DII categories are presented as means with standard deviations (SD) for quantitative variables and as frequencies and percentages for categorical variables.

Associations between the E-DII and severe NAFLD were first investigated using Cox proportional hazard models by categories of the E-DII. Individuals in the very/moderately anti-inflammatory category were used as the referent. The results are reported as hazard ratios (HR) and their 95% confidence intervals (95% CIs). Duration of follow-up was used as the time-dependent variable.

**Fig. 1:**
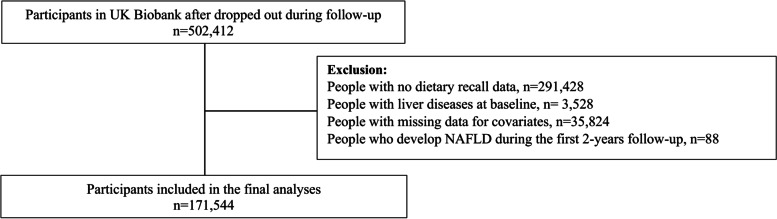
Diagram of participants included in the analyses

Nonlinear associations between the continuous E-DII score and severe NAFLD were investigated using penalised cubic splines fitted in Cox proportional hazard models. The penalised spline is a variation of the basis spline, which is less sensitive to knot numbers and placements than restricted cubic splines [45]. The mean value of the score was used as a referent group (mean value =  − 0.28) in the splines. The proportional hazard assumption was checked using Schoenfeld residuals. Using the Expert Panel Consensus Statement [39], participants with other liver disease or alcohol/drug use disorders at or before baseline (*n* = 3528) were excluded. In addition, all analyses were conducted using 2-year landmark analyses, excluding all participants who experienced events within the first 2 years of follow-up (*n* = 88) (Fig. 1). This approach minimised the effect of reverse causality.

Analyses were adjusted for confounding factors based on previous literature, using the following four models: model 0 was unadjusted. Model 1 was adjusted for sociodemographic factors (age, sex, deprivation and ethnicity). Model 2 was adjusted as per model 1 but additionally included health-related factors (the individual components of the metabolic syndrome [central obesity, hyperglycaemia/diabetes, hypertension/high blood pressure, low HDL and high triglyceride] and self-reported inflammatory diseases). Model 3 was additionally adjusted for lifestyle factors (smoking and physical activity). These covariates were selected based on existing evidence as they are related to the exposure and outcome [3, 6–8].

Finally, to investigate whether the association between the continuous E-DII score and severe NAFLD differed by population groups, we tested for interactions and stratified the analyses by age (≥ and < 60 years), sex (men and women), deprivation (Townsend score ≤ and > the median), inflammatory disease (no or yes) and smoking status (never and previous/current). In this case, analyses were adjusted for the covariates included in model 3 when these were not included as a subgroup.

Stata 17 and R 3.6.1 were used to perform the analyses. A *p*-value below 0.05 was considered statistically significant.

## Results

After excluding people with other liver diseases or alcohol/drug use disorders as well as those with missing data for the E-DII score and covariates, 171,544 participants with complete data available were included in the analyses (Fig. 1). Over a median follow-up of 10.2 years (interquartile range: 9.58 to 10.9 years), 1489 (0.9%) participants developed severe NAFLD.

The baseline characteristics of the study participants, broken down by the E-DII categories, are shown in Table 1. Overall, participants in the very/moderately anti-inflammatory category were older, more likely to be women, less deprived and more likely to have never smoked and to walk for pleasure, compared to those in the neutral or very/moderately pro-inflammatory category. Moreover, participants in the very/moderately anti-inflammatory category had a lower prevalence of central obesity and high triglycerides as well as a lower prevalence of low HDL concentrations (Table 1).

**Table 1:** Baseline cohort characteristics

	**Total**	**Very/moderately anti-inflammatory**	**Neutral**	**Very/moderately pro-inflammatory**
*n* (%)	171,544 (100)	52,377 (30.5)	88,326 (51.5)	30,841 (18.0)
Baseline age (years), mean (SD)	56.1 (8.0)	56.9 (7.7)	56.0 (8.0)	55.0 (8.2)
Sex,* n* (%)				
Women	93,963 (54.8)	34,038 (65.0)	46,256 (52.4)	13,669 (44.3)
Men	77,581 (45.2)	18,339 (35.0)	42,070 (47.6)	17,172 (55.7)
Deprivation index, mean (SD)	− 1.59 (2.86)	− 1.68 (2.81)	− 1.64 (2.83)	− 1.29 (2.99)
Ethnicity, *n* (%)				
White	164,257 (95.8)	50,057 (95.6)	84,830 (96.0)	29,370 (95.2)
Others	7287 (4.2)	2320 (4.4)	3496 (4.0)	1471 (4.8)
Smoking status, *n* (%)				
Never	97,340 (56.7)	30,482 (58.2)	50,434 (57.1)	16,424 (53.3)
Previous	61,002 (35.6)	19,157 (36.6)	31,325 (35.5)	10,520 (34.1)
Current	13,202 (7.7)	2738 (5.2)	6567 (7.4)	3897 (12.6)
**Type of physical activity**				
Walking for pleasure (not as a means of transport)	127,962 (74.6)	41,321 (78.9)	65,916 (74.6)	20,725 (67.2)
Other exercises (e.g. swimming, cycling, keep fit, bowling)	21,411 (12.5)	6067 (11.6)	11,191 (12.7)	4153 (13.5)
Strenuous sports	1367 (0.8)	319 (0.6)	727 (0.8)	321 (1.0)
Light DIY (e.g. pruning, watering the lawn)	10,148 (5.9)	2313 (4.4)	5234 (5.9)	2601 (8.4)
Heavy DIY (e.g. weeding, lawn mowing, carpentry, digging)	3282 (1.9)	747 (1.4)	1656 (1.9)	879 (2.8)
None of the above	7315 (4.3)	1595 (3.0)	3576 (4.0)	2144 (7.0)
Prefer not to answer	59 (< 0.1)	15 (< 0.1)	26 (< 0.1)	18 (< 0.1)
Inflammatory diseases (yes), *n* (%)	21,373 (12.5)	6436 (12.3)	10,899 (12.3)	4038 (13.1)
Hyperglycaemia/diabetes (yes), *n* (%)	24,556 (14.3)	7441 (14.2)	12,675 (14.3)	4440 (14.4)
Low HDL (yes), *n* (%)	30,446 (17.8)	8981 (17.2)	15,115 (17.1)	6350 (20.6)
High triglycerides (yes), *n* (%)	64,154 (37.4)	17,629 (33.7)	33,430 (37.8)	13,095 (42.5)
Central obesity (yes), *n* (%)	51,353 (29.9)	14,359 (27.4)	26,220 (29.7)	10,774 (34.9)
High blood pressure/hypertension (yes), *n* (%)	116,420 (67.9)	35,313 (67.4)	60,046 (68.0)	21,061 (68.3)

Descriptive characteristics by E-DII categories are presented as means with SD for quantitative variables and as frequencies and percentages for categorical variables. *n* Number, *SD* Standard deviation

Associations between the E-DII categories and severe NAFLD are shown in Table 2. In the unadjusted and minimally adjusted models, individuals in the very/moderately pro-inflammatory category had 1.54- (95% CI: 1.33 to 1.78) and 1.49-times (95% CI: 1.28 to 1.72) higher risk of severe NAFLD, respectively, than those in the very/moderately anti-inflammatory group (models 0 and 1). The magnitude of the associations was somewhat attenuated when the models were further adjusted for health-related factors (HR: 1.24 [1.08 to 1.44]). The association was further attenuated after adjusting for lifestyle factors but remained significant (HR: 1.19 [1.03 to 1.38]). Although no significant associations were identified for the neutral group compared to their counterparts, there was a statistically significant trend across the three categories (Table 2). Individual associations of each covariate included in these analyses can be found in Additional file 1: Table S1.

**Table 2:** Associations between the E-DII categories and severe NAFLD

	**Very/moderately anti-inflammatory**	**Neutral**		**Very/moderately pro-inflammatory**		**Trend**	
**Incident severe NAFLD**	HR (95%CI)	HR (95% CI)	*p*-value	HR (95% CI)	*p*-value	HR (95% CI)	*p*-value
Model 0	1.00 (Ref.)	1.11 (0.98; 1.26)	0.087	1.54 (1.33; 1.78)	< 0.001	1.24 (1.15; 1.33)	< 0.001
Model 1	1.00 (Ref.)	1.11 (0.98; 1.26)	0.097	1.49 (1.28; 1.72)	< 0.001	1.22 (1.13; 1.31)	< 0.001
Model 2	1.00 (Ref.)	1.04 (0.92; 1.18)	0.489	1.24 (1.08; 1.44)	0.003	1.11 (1.03; 1.20)	0.004
Model 3	1.00 (Ref.)	1.03 (0.91; 1.16)	0.649	1.19 (1.03; 1.38)	0.020	1.09 (1.01; 1.17)	0.024

Associations between E-DII and severe NAFLD were investigated by E-DII categories and the continuous score using Cox proportional hazard models. Individuals in the very/moderate anti-inflammatory category were used as the referent. All analyses were performed using a 2-year landmark analysis, excluding participants who experienced events within the first 2 years of follow-up and those with liver disease or alcohol/drug use disorder at baseline. Model 0 was unadjusted. Model 1 was adjusted for age, sex, deprivation and ethnicity. Model 2, as per model 1 but additionally for the components of the metabolic syndrome (central obesity, high glycaemia/diabetes, high blood pressure/hypertension, low HDL and high triglyceride) and inflammatory diseases. Model 3, as per model 2 and also for smoking and physical activity. A *p*-value below 0.05 was considered statistically significant

**Fig. 2:**
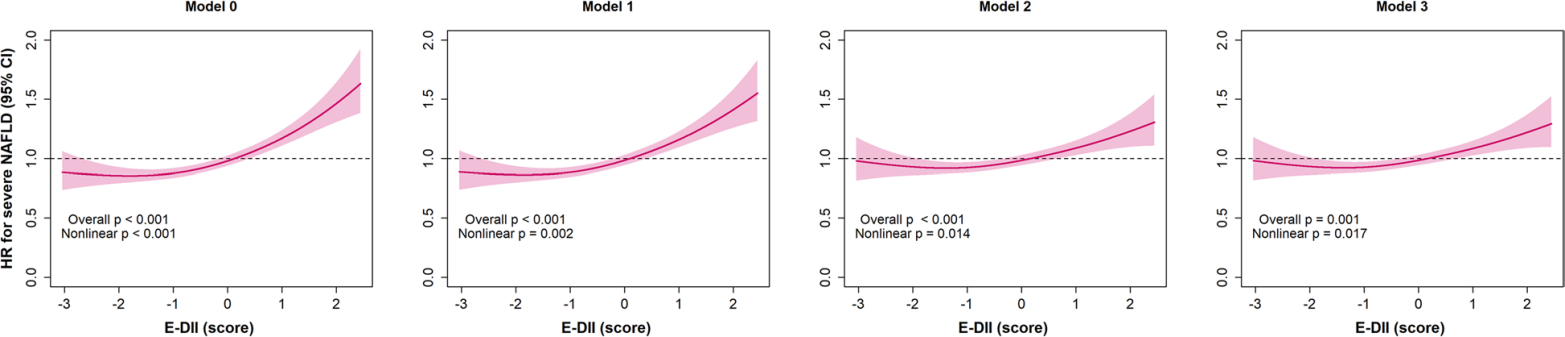
Association between the E-DII and severe NAFLD. A nonlinear association between the E-DII and severe NAFLD was investigated using penalised cubic splines fitted in Cox proportional hazard models. Analyses were performed using the same information reported in Table 2

A nonlinear association between the continuous E-DII score and severe NAFLD is shown in Fig. 2. Overall, a higher E-DII score was associated with a higher severe NAFLD risk in all models (models 0 to 3). In the minimally adjusted model, the highest risk was observed among individuals with a score ≥ 2, who had ~ 60% higher risk of severe NAFLD than those with the mean E-DII value. The risk was attenuated after adjusting for other confounders (model 3) but remained significant (*p*-overall = 0.001). Additionally, there was evidence of nonlinearity between the E-DII score and severe NAFLD in all models, with a J-shaped association whereby the lowest NAFLD risk was observed among participants with an E-DII score between − 2 and − 1 (Fig. 2).

Finally, no significant interactions were identified; hence, the associations were broadly consistent across subgroups (Additional file 1: Fig. S1).

## Discussion

Among 171,544 UK Biobank participants, a higher risk of incident severe NAFLD was observed in individuals who consumed a more pro-inflammatory diet, independent of confounders. The association appears to be nonlinear. The minimum risk of severe NAFLD was found among individual who had a slightly anti-inflammatory diet, and there was no evidence that a more anti-inflammatory diet would result in lower risk. Considering the increasing prevalence of NAFLD and the urgent need to promote modifiable risk factors that can slow its development, improving the quality of the diet should be a major public health priority.

To our knowledge, only one previous study investigated the association between anti-inflammatory diets and NAFLD risk among 3042 Greek adults participating in the ATTICA prospective study [31]. Using a methodology that followed the DII as previously published by Shivappa et al. [21], Tyrovolas et al. developed a modified version of the original DII named “D-AII”, which was then split into tertiles [31]. Compared to individuals in the lowest tertile (more pro-inflammatory), those with diets in the highest tertile (more anti-inflammatory) had lower odds of NAFLD (odds ratio [OR_for the triglyceride-glucose marker_]: 0.33 [0.24 to 0.47]). In contrast to our study, Tyrovolas et al. used logistic regression rather than time-to-event analysis and were therefore unable to take into account temporal associations in the data and loss to follow-up and competing risk; for example, due to death from other causes. Furthermore, the investigators did not have access to NAFLD diagnoses based on ultrasound or magnetic resonance imaging and, therefore, used hepatic markers, such as triglyceride-glucose index, as proxy measures. Finally, whilst Tyrovolas et al. included a long list of confounders in their analyses, they did not include inflammatory diseases that may impact people’s diet [31]. Therefore, our study responds to the research question and is also the first to investigate both the linear and nonlinear anti-inflammatory role of diet in the UK population.

Regarding the association between diet and NAFLD, other prospective studies have investigated this association using different approaches: either other diet indices or some of the individual elements included in the DII [13–19]. For instance, Zhang et al., using data from the Tianjin Chronic Low-Grade Systematic Inflammation and Health Cohort Study, identified that the consumption of ultra-processed food [17], soft drinks [16], sugar-rich dietary patterns and animal food patterns were associated with a higher risk of NAFLD [13]. In these studies, the highest risk was observed in individuals who drank ≥ 4 servings/week of soft drinks compared to those who drank < 1 serving/week (HR: 1.59 [1.07 to 2.37]) [16]. Similar results were identified in Iranian adults, where adherence to a Mediterranean diet was inversely associated with NAFLD (OR: 0.64 [0.52 to 0.78]) [19], and in Korean adults, where a higher consumption of fruit and vegetables was associated with a lower NAFLD risk (risk ratio [RR] fruit: 0.77 [0.62 to 0.96]; RR vegetable: 0.71 [0.56 to 0.88]) [14]. Therefore, a healthy diet might protect against NAFLD development irrespective of the specific diet approach.

Pro-inflammatory dietary nutrients — such as total, saturated and *trans* fats — might contribute to the pathogenesis of NAFLD by promoting low-grade systemic inflammation [18]. Overconsumption will also contribute to a higher deposition of triglycerides in the liver. The latter will induce insulin resistance, hyperinsulinemia, liver inflammation, oxidative stress, mitochondrial dysfunction, imbalanced pro-inflammatory cytokines and fibrosis — which are all associated with NAFLD [9]. Furthermore, an unbalanced diet might also contribute to obesity, type 2 diabetes and metabolic syndrome, diseases directly related to NAFLD development [5].

Finally, the progression of NAFLD may have devastating consequences because it can lead to hepatocellular carcinoma, which is aggressive and usually rapidly fatal [2, 46]. As there is no approved treatment for the disease, lifestyle modification remains the key strategy to lower the risk of NAFLD development and progression. Nonetheless, achieving and maintaining healthier dietary patterns remains challenging [47], especially those associated with controlling body weight. Therefore, the findings from this study are directly relevant to this important and growing global health problem.

### Strength and limitations

UK Biobank allowed the investigation of our research question in a single, large and well-characterised general population cohort of middle-aged and older adults. In addition, analyses were adjusted for a diverse list of confounders, including the common drivers of NAFLD and inflammatory diseases. Likewise, we were able to assess the linearity of the association and whether it was consistent across subgroups. Also, a major driver of potential information bias, knowledge of disease status, was obviated completely by ascertaining outcomes from routine administrative databases. Despite its strengths, this study also has limitations. First, ascertainment of NAFLD was based on hospital admission and death records and was, therefore, restricted to more advanced or severe cases of the disease since they had to be hospitalised or die due to NAFLD. Moreover, even if electronic health records allow the identification of a large amount of data, the use of other biomarkers — such as FIB-4 (Fibrosis-4 index for liver fibrosis), NFS (NAFLD Fibrosis Score) or APRI (aspartate aminotransferase-to-platelet ration index) — might also be used as predictors of disease severity. Unfortunately, these biomarkers were not available in the UK Biobank study, or the information used to estimate them was only available at baseline assessment. Second, although we included those confounding factors that were considered relevant and for which we had data, unmeasured or residual confounding is possible. Third, the E-DII was created from self-reported data, which may result in some inaccuracies. We handled misreporting by excluding people with unfeasible energy intake based on Henry’s equation. However, even if the average of five 24-h recalls was used to create the E-DII score, diet is subjected to recall and misclassification bias, and diet might have changed over time. Moreover, the number of participants who had repeated dietary data was less than 20% of the original cohort. Hence, results need to be considered with caution. However, there is no reason to suspect a systematic error in relation to future NAFLD and, therefore, concern about the disease-differential recall or misclassification bias. We minimised potential reverse causation by using a 2-year landmark analysis. Fourth, the instrument in this study was able to map only 18 food parameters, which is much smaller than the average of about 27 to 30 available in most studies [30]. As most of the missing food parameters are anti-inflammatory, this would tend to create an asymptote around low E-DII values and compression of the overall distribution that may influence results, including precision of estimates of association. Fifth, associations observed in an observational study cannot be assumed to infer causality. Sixth, we did not conduct analyses by specific ethnicity due to the small number of participants in the non-white category (7287, representing only 4.2% of the total included population). Finally, UK Biobank is not representative of the UK population in relation to lifestyle and prevalent diseases. Therefore, whilst risk estimates can be generalised [48], summary statistics such as prevalence and incidence should not [49].

## Conclusions

In conclusion, pro-inflammatory diets were associated with a higher risk of severe NAFLD independent of confounders, including the metabolic syndrome components. Since there is no recommended treatment for the disease, improving diet quality could be a priority in reducing the risk of NAFLD. Future studies are needed to prove causation and to investigate diets that — along with other effective interventions — might reduce the burden of NAFLD.

## Supplementary Information

**Additional file 1: Figure S1.** Association between the E-DII and severe NAFLD by subgroups. **Table S1.** Associations between DII categories, each confounder and severe NAFLD.

## Data Availability

All UK Biobank information is available online on the webpage www.ukbiobank.ac.uk/. Data access is available through applications. This research was conducted using the application number 7155.

## Abbreviations

D-AII: Dietary Anti-Inflammation Index
DII: Diet Inflammatory Index
E-DII: Energy-adjusted Diet Inflammatory Index
HES: Health Episode Statistics
HR: Hazard ratio
ICD: International Classification of Diseases
IQR: Interquartile range
NAFLD: Non-alcoholic fatty liver disease
NASH: Non-alcoholic steatohepatitis
NHS: National Health Service
PHE: Public Health England
SD: Standard deviations
SMR01: Scottish Morbidity Records
