# Supplementary material for: Associations between an inflammatory diet index and severe non-alcoholic fatty liver disease: a prospective study of 171,544 UK Biobank participants
Source: BMC Med. 2023 Apr 3;21:123. doi: 10.1186/s12916-023-02793-y (PMC10071692; doi:10.1186/s12916-023-02793-y)
Supplement: Supplementary file 1 — Additional file 1: Figure S1. Association between the E-DII and severe NAFLD by subgroups. Table S1. Associations between DII categories, each confounder and severe NAFLD. [file 12916_2023_2793_MOESM1_ESM.docx]

**Additional file 1:**

**Associations between an inflammatory diet index and severe non-alcoholic fatty liver disease: a prospective study of 171,544 UK Biobank participants**

[**Figure S1. Association between the E-DII and severe NAFLD by subgroups** 2](#_Toc126054642)

[**Table S1. Associations between E-DII categories, each confounder and severe NAFLD** 4](#_Toc126054643)


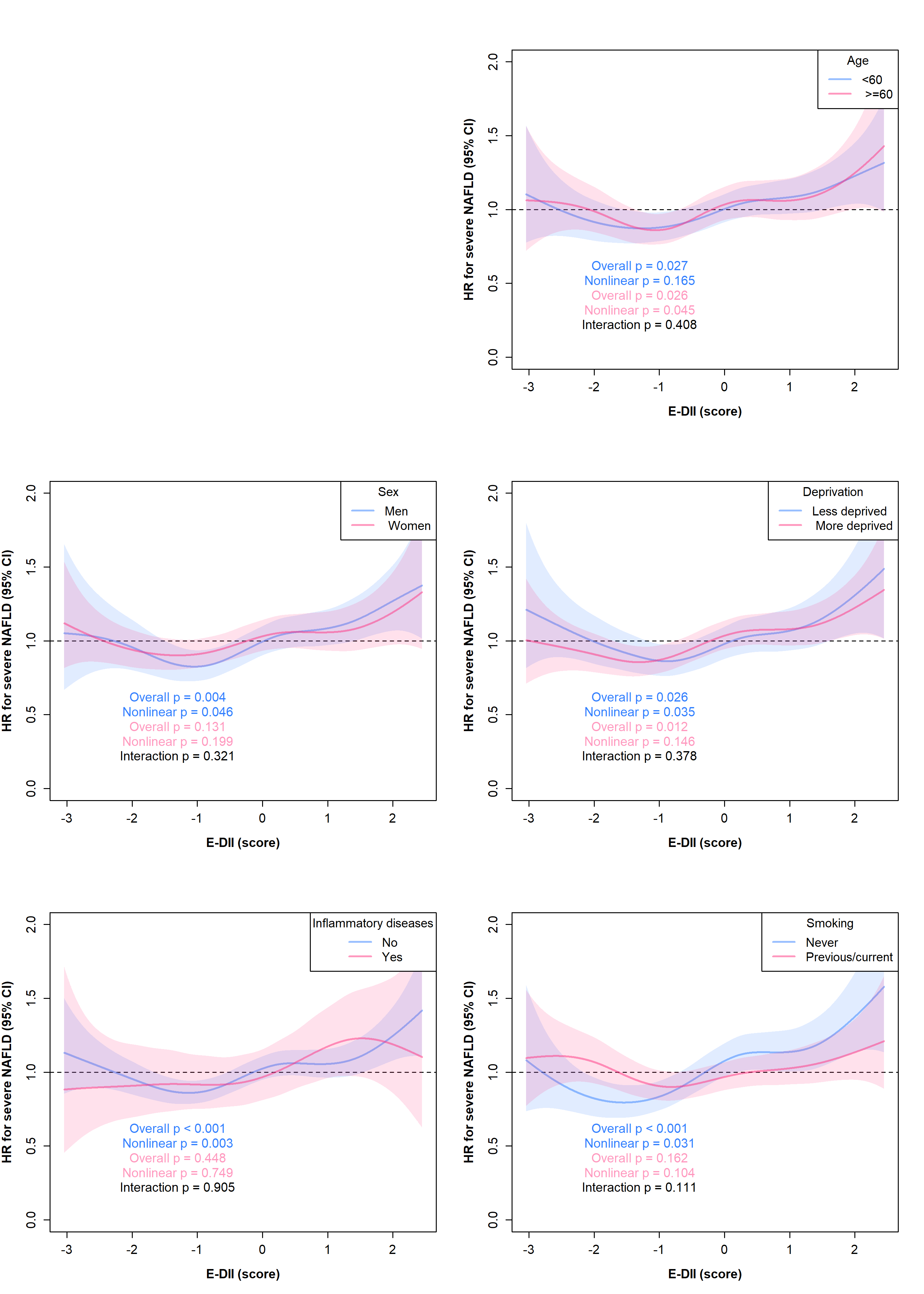


# **Figure S1. Association between the E-DII and severe NAFLD by subgroups**

Nonlinear associations between the E-DII and severe NAFLD were investigated using penalised cubic splines fitted in Cox proportional hazard models. All analyses were performed using a 2-year landmark analysis, excluding participants who experienced events within the first two years of follow-up and those with liver disease or alcohol/drug use disorder at baseline. Analyses were adjusted for age, sex, deprivation, ethnicity, the components of the metabolic syndrome (central obesity, high glycaemia/diabetes, high blood pressure/hypertension, low HDL and high triglyceride), inflammatory diseases, smoking and physical activity when these were not included as a subgroup.

# **Table S1. Associations between E-DII categories, each confounder and severe NAFLD**

| **Variable** | **HR (95% CI)** | **p-value** |
| --- | --- | --- |
| **E-DII categories** |  |  |
| Very/moderately anti-inflammatory | 1.00 (Ref.) |  |
| Neutral | 1.03 (0.91; 1.16) | 0.649 |
| Very/moderately pro-inflammatory | 1.19 (1.03; 1.38) | 0.020 |
| **Age*** | 1.00 (0.99; 1.01) | 0.884 |
| **Sex** |  |  |
| Women | 1.00 (Ref.) |  |
| Men | 1.11 (0.99; 1.23) | 0.066 |
| **Deprivation index*** | 1.08 (1.06; 1.10) | <0.001 |
| **Ethnicity** |  |  |
| White | 1.00 (Ref.) |  |
| Others | 1.00 (0.78; 1.27) | 0.982 |
| **Smoking status** |  |  |
| Never | 1.00 (Ref.) |  |
| Previous | 1.26 (1.13; 1.40) | <0.001 |
| Current | 1.20 (1.00; 1.44) | 0.048 |
| **Type of physical activity** |  |  |
| Walking for pleasure | 1.00 (Ref.) |  |
| Other exercises | 1.31 (1.13; 1.52) | <0.001 |
| Strenuous sports | 0.98 (0.51; 1.89) | 0.956 |
| Light DIY | 1.49 (1.25; 1.77) | <0.001 |
| Heavy DIY | 1.12 (0.80; 1.55) | 0.512 |
| None of the above | 1.44 (1.20; 1.74) | <0.001 |
| Prefer not to answer | - | - |
| **Health status** |  |  |
| Inflammatory diseases (no) | 1.00 (Ref.) |  |
| Inflammatory diseases (yes) | 1.27 (1.10; 1.45) | 0.001 |
| Hiperglycaemia/diabetes (no) | 1.00 (Ref.) |  |
| Hiperglycaemia/diabetes (yes) | 1.34 (1.18; 1.51) | <0.001 |
| Low HDL (no) | 1.00 (Ref.) |  |
| Low HDL (yes) | 1.75 (1.57; 1.96) | <0.001 |
| High triglycerides (no) | 1.00 (Ref.) |  |
| High triglycerides (yes) | 1.39 (1.24; 1.55) | <0.001 |
| Central obesity (no) | 1.00 (Ref.) |  |
| Central obesity (yes) | 3.02 (2.68; 3.39) | <0.001 |
| High blood pressure /hypertension (no) | 1.00 (Ref.) |  |
| High blood pressure /hypertension (yes) | 1.39 (1.21; 1.59) | <0.001 |

All analyses were performed using a 2-year landmark analysis, excluding participants who experienced events within the first two years of follow-up and those with liver disease or alcohol/drug use disorder at baseline. Analyses were adjusted for DII categories, age, sex, deprivation, ethnicity, the components of the metabolic syndrome (central obesity, high glycaemia/diabetes, high blood pressure/hypertension, low HDL and high triglyceride), inflammatory diseases, smoking and physical activity. A p-value below 0.05 was considered statistically significant. *Continuous variable. – unpowered analysis.
